# Perceptions on Burnout and the Medical School Learning Environment of Medical Students Who Are Underrepresented in Medicine

**DOI:** 10.1001/jamanetworkopen.2022.0115

**Published:** 2022-02-23

**Authors:** Jamieson M. O’Marr, Shin Mei Chan, Lake Crawford, Ambrose H. Wong, Elizabeth Samuels, Dowin Boatright

**Affiliations:** 1 Yale School of Medicine, New Haven, Connecticut; 2 Department of Emergency Medicine, Yale School of Medicine, New Haven, Connecticut; 3 Department of Emergency Medicine, Alpert Medical School of Brown University, Providence, Rhode Island

## Abstract

**Importance:**

Burnout is a highly prevalent issue among medical trainees, but there has been limited research characterizing burnout specifically among medical students from groups who are underrepresented in medicine (URIM).

**Objective:**

To assess the association between components of the medical school learning environment and burnout among medical students who are URIM vs those who are not.

**Design, Setting, and Participants:**

This retrospective cross-sectional survey study evaluated responses of allopathic medical students graduating from all US allopathic medical schools in 2016 and 2017 to the American Medical Colleges Graduation Questionnaire. Analysis was completed between December 1, 2019, and July 1, 2020.

**Exposures:**

Self-identification as a medical student who is URIM.

**Main Outcomes and Measures:**

Self-reported measures of medical student overall, disengagement, and exhaustion-related burnout using the Oldenburg Burnout Inventory for Medical Students.

**Results:**

The American Medical Colleges Graduation Questionnaire had an 81% response rate, yielding 26 567 complete participant responses that were included the analysis. A total of 13 645 individuals (51.4%) were male, and 3947 (14.9%) identified as URIM (ie, Alaska Native, Black, Hispanic/Latinx, Native American, and/or Pacific Islander). Medical students who are URIM reported modestly higher levels of exhaustion-related burnout (mean [SD], 11.84 [3.62] vs 11.48 [3.61]; *P* < .001) and modestly lower mean burnout scores associated with disengagement (mean [SD], 9.24 [3.56] vs 9.36 [3.58]; *P* = .047). Medical students who are URIM also reported marginally less favorable student-faculty interactions in the learning environment (mean [SD], 14.09 [3.45] vs 14.29 [3.35]; *P* < .001). Medical students who are URIM were more likely to be in the top quartile of those who experienced exhaustion-related burnout (odds ratio, 1.19 [95% CI, 1.09-1.29]) but less likely to be in the top quartile for disengagement (odds ratio, 0.87 [95% CI, 0.80-0.94]). Regardless of URIM status, those who reported learning environment scores in the bottom quartile were more likely to experience higher rates of burnout as were those who experienced at least 1 episode of discrimination.

**Conclusions and Relevance:**

This survey study found that medical students who are URIM had a higher risk for exhaustion-related burnout. This burnout is likely multifactorial and could represent a resiliency or survival bias, the burden of increased responsibility, and/or recurrent discrimination. The learning environment can play a key role in mitigating burnout in both medical students who are URIM and those who are not and is deserving of further research.

## Key Points

**Question:**

How are medical students’ racial and ethnic identities and perceptions of the medical school learning environment associated with burnout?

**Findings:**

In this survey study of 26 567 graduating medical students, medical students who are underrepresented in medicine (URIM) reported experiencing modestly higher levels of exhaustion-related burnout and were at increased odds of experiencing burnout if they experienced racial discrimination during training.

**Meaning:**

The medical school learning environment influences burnout among all medical students, but students who are URIM experience higher rates of discrimination and exhaustion-related burnout; reducing discrimination and identifying and addressing relevant components of the learning environment must be prioritized to prevent burnout in medical students who are URIM.

## Teaser

This survey study assesses the association between components of the medical school learning environment and burnout among medical students who are underrepresented in medicine vs those who are not.

## Introduction

Burnout is a common phenomenon among medical professionals^1,2^ and can lead to emotional exhaustion, depression, and suicidal ideation.^3,4,5,6,7^ In addition to negative effects on clinicians, burnout significantly impairs patient care and contributes to decreased patient satisfaction and interactions, quality of medical care, and medical errors.^8^ Burnout in medicine can occur as early as during medical school training and even prior.^9^ Among medical students, burnout has been reported to affect up to 50% of trainees.^5,10^ Recent research has focused on identifying factors such as adverse medical school experiences, including mistreatment, that contribute to burnout among medical students.^11,12^ Additionally, sociodemographic factors have been explored, and students identifying as sexual or gender minorities, including lesbian, gay, bisexual, transgender, queer, or intersex individuals, have been shown to experience increased rates of burnout owing to mistreatment.^13^

More recent research has begun to explore burnout among medical students who are underrepresented in medicine (URIM) (ie, those that identify as Alaska Native, Black, Hispanic/Latinx, Native American, and/or Pacific Islander).^14^ Medical students who are URIM experience medical training more negatively compared with peers who are not URIM owing to structural racism and bias that exists in classroom and health care delivery settings.^14,15^ They are also at greater risk of depression during medical school owing to poor diversity climates, alienation, and lack of peer and faculty support.^6,16,17,18^ Additionally, students who are URIM experience high rates of mistreatment, which is associated with medical student burnout, including public humiliation and discriminatory remarks.^11,19^ However, the degree to which these experience contribute to burnout among medical students who are URIM is not known.^6,20^

This study assessed how the medical school learning environment (ie, the social, mental, and physical space for medical students) contributes to burnout among medical students who are URIM.^21^ Previous work has demonstrated that a poor medical school learning environment can lead to medical student burnout.^4,21,22,23,24^ Two important components of the learning environment, faculty interactions and emotional climate, strongly influence student medical school experiences. Other studies have shown that faculty may play important roles in mitigating burnout by providing learning autonomy and support to students.^22^ On the other hand, the emotional climate describes medical students’ affective response to the learning environment. Overall, the learning environment likely affects long-term well-being, given that distress, mistreatment, and poor emotional health can lead to burnout.^11,14,25^ The differential perceptions concerning the faculty interactions and emotional climate of the learning environment among medical students by race and ethnicity remain poorly understood and have not been studied on a national level.

In this study, these 2 important elements of the learning environment measured by a national questionnaire administered to all graduating allopathic US medical students were analyzed. Additionally, as prior research has demonstrated that students who are URIM experience disproportionate levels of racial mistreatment and discrimination in the medical school learning environment (compared with peers who are not URIM), this study further assesses the role that discrimination plays in burnout.^14,19^

## Methods

Responses to the 2016 and 2017 Association of American Medical Colleges Graduation Questionnaire (AAMC GQ) were analyzed. The AAMC GQ is administered yearly to graduating medical students from accredited allopathic medical schools in the US, representing 38 197 students in 2016 and 2017. This survey adheres to the American Association for Public Opinion Research (AAPOR) reporting guideline of named persons by reporting returned questionaries and eligible nonresponses (American Association for Public Opinion Research code 1.0 and 2.0).^26^ It gathers information regarding demographics, preclinical and clinical educational experiences, negative behaviors experienced, future career plans, and finances.^27^ Additionally, the AAMC GQ includes students’ reports of burnout and students’ perceptions of medical school student-faculty interactions and emotional climate. This study was approved by the Yale University Institutional Review Board. The AAMC obtained written consent from participants, and deidentified AAMC data were used for this study.

### Student Demographics and Medical School Characteristics

For this analysis, we defined medical students who self-identified as Black, Hispanic, Native American/Alaskan Native, and/or Pacific Islander as URIM.^28^ Other demographic information included age (AAMC categories: younger than 24 years, 24-26 years, 27-29 years, 30-32 years, or 33 years or older), sex (male or female), marital status, and presence of medical loans. Medical school characteristics included type of school ownership (public vs private).

### Burnout Measures

Burnout was measured using the Oldenburg Burnout Inventory for Medical Students (OLBI-MS),^29,30^ which surveys symptoms of burnout along 2 constructs: exhaustion and disengagement (eTable in the Supplement). The exhaustion subscale measures the mental and physical exhaustion experienced amid the stressors of medical school, whereas the disengagement subscale represents students’ level of interest and engagement with medical school work.^31^ Relevant questions were reverse coded to create a summary score where higher scores represent higher levels of burnout. The OLBI-MS has a maximum score of 48 with the 2 constructs of exhaustion and disengagement each with a maximum score of 24 points.

### Medical Student Learning Environment

The Medical Student Learning Environment Survey (MSLES) questions in the AAMC GQ^32^ focus on 2 domains: emotional climate (maximum score, 15) and student-faculty interaction (maximum score, 20) (eTable in the Supplement). Questions about the emotional climate pertain to student feelings of accomplishment, self-value, and academic confidence.^33^ Student-faculty interaction questions focus on how students perceive faculty support at their medical schools. Relevant questions were transformed via reverse coding to calculate overall MSLES scores for each respondent. Higher summation scores represented more positive perceptions of the learning environment.

### Student Discrimination

We created a binary discrimination event variable for having ever experienced mistreatment based on race or ethnicity using 3 survey questions regarding experiences of mistreatment based on race or ethnicity. These questions asked students to recall how frequently they were denied opportunities, received lower evaluations, or were subjected to offensive remarks because of their race or ethnicity on a scale of never, once, occasionally, or frequently. Any student who reported at least 1 event (once, occasionally, or frequently) for 1 or more of the questions was included as having experienced discrimination.

### Statistical Analysis

Overall burnout, burnout subscales, and MSLES scores were stratified into quartiles based on numeric summary score for each construct and assessed as categorical variables. Quartiles were used to give meaning to the ranges of scores relative to total student respondents. Students in the highest quartiles for burnout scores represent the students at highest risk for burnout. The higher quartiles of MSLES represent more positive student perceptions about their school learning environment. Differences in demographic characteristics as well as differences between MSLES, overall burnout, and subscale burnout quartiles between students who are URIM and thoes who are not were assessed using χ^2^ tests. Two-tailed, unpaired *t* tests for continuous variables were used to compare the mean values for MSLES and burnout components between students who are URIM and those who are not. Statistical significance was set at *P* < .05.

Logistic regression analyses were conducted to model the association of URIM status on the likelihood of a student having scores in the top quartile for overall burnout scale and the subscales of disengagement and exhaustion. Our model was created using a priori subject level-expertise and existing literature. MSLES scores (emotional climate and student-faculty interaction) were included based on previous research.^19^ An interaction term between URIM status and MSLES score was included to explore a potential interaction between these variables. The fully adjusted model included participant demographics, experiences, and medical student characteristics variables (sex, age, marital status, student loans, discrimination events, and school type) that could or have been shown to contribute to burnout symptoms.^3,13,34,35^ Odds ratios (ORs) and 95% CIs were calculated to quantify the strength of observed associations. All statistics were performed using R version 3.6.3 (R Foundation). Analysis took place from December 1, 2019, to July 1, 2020.

## Results

A total of 30 651 students responded to the AAMC-GQ (response rate, 80.7%). We excluded students who did not complete OLBI-MS and/or MSLES questions (n = 4084) (eFigure in the Supplement). The rates of exclusion were similar between students who are URIM and those who are not (738 of 4685 [15.8%] vs 3346 of 25 966 [12.9%]). After all exclusions were made, 26 567 participant responses were analyzed (Table 1). Of these participants, 3947 (14.9%) identified as URIM. As shown in Table 1, students who are URIM were more likely to be female (2167 [54.9%] vs 10 755 [47.5%]; *P* < .001). They were also more likely to have attended a private medical school (1652 [41.9%] vs 8756 [38.7%]; *P* < .001) and have a medical school loan burden (3271 [82.9%] vs 16 000 [70.7%]; *P* < .001). Finally, students who are URIM were more likely to have experienced events of discrimination during their time in medical school (748 [19.0%] vs 1628 [7.2%]; *P* < .001).

**Table 1.** Study Participant Characteristics From the Medical Student Learning Environment Survey Stratified by URIM Status

Characteristic	No. (%)			*P* value
	Total (N = 26 567)	URIM (n = 3947)	Not URIM (n = 22 620)	
Sex				
Male	13 645 (51.4)	1780 (45.1)	11 865 (52.5)	<.001^a^
Female	12 922 (48.6)	2167 (54.9)	10 755 (47.5)	
Age category, y				
≤23	89 (0.3)	14 (0.4)	75 (0.3)	<.001^a^
24-26	10 950 (41.2)	1425 (36.1)	9525 (42.1)	
27-29	10 948 (41.2)	1680 (42.6)	9268 (41.0)	
30-32	3057 (11.5)	525 (13.3)	2532 (11.2)	
≥33	1523 (5.7)	303 (7.7)	1220 (5.4)	
Medical loans				
Yes	19 271 (72.5)	3271 (82.9)	16 000 (70.7)	<.001^a^
No	6980 (26.3)	614 (15.6)	6366 (28.1)	
Decline to answer	316 (1.2)	62 (1.6)	254 (1.1)	
School ownership				
Private	10 408 (39.2)	1652 (41.9)	8756 (38.7)	<.001^a^
Public	16 159 (60.8)	2295 (58.1)	13 864 (61.3)	
Marital status				
Single	19 230 (72.4)	2981 (75.5)	16 249 (71.8)	<.001^a^
Married/partnered	6688 (25.2)	816 (20.7)	5872 (26.0)	
Divorced/separated/widowed	305 (1.1)	73 (1.8)	232 (1.0)	
Decline to answer	344 (1.3)	77 (2.0)	267 (1.2)	
Discrimination				
No discrimination	23 879 (89.9)	3148 (79.8)	20 731 (91.6)	<.001^a^
≥1 Discrimination episodes	2376 (8.9)	748 (19.0)	1628 (7.2)	
Decline to answer	312 (1.2)	51 (1.3)	261 (1.2)	

Abbreviation: URIM, underrepresented in medicine.

^a^ *P* value represents Pearson χ^2^ test.

Students who are URIM had modestly higher mean overall burnout (mean [SD], 21.08 [6.58] vs 20.84 [6.64]; *P = *.03) and exhaustion (mean [SD], 11.84 [3.62] vs 11.48 [3.61]; *P* < .001) but lower mean disengagement scores (mean [SD], 9.24 [3.56] vs 9.36 [3.58]; *P* = .047) compared with their counterparts who are not URIM (Table 2). Students who are URIM reported slightly more favorable medical school emotional climate (mean [SD], 9.80 [3.29] vs 9.62 [3.21]; *P* = .002) but less favorable student-faculty interactions (mean [SD], 14.09 [3.45] vs 14.29 [3.35]; *P* < .001) compared with their counterparts who are not URIM (Table 2).

**Table 2.** Oldenburg Burnout and Learning Environment Summary Scores by URIM Status^a^

Characteristic	No. (%)		*P* value
	URIM	Not URIM	
**Burnout**			
Overall burnout			
Mean (SD)	21.08 (6.58)	20.84 (6.64)	.03^b^
Quartile counts			
Highest	870 (22.0)	4864 (18.3)	.30^c^
Second	963 (24.4)	5351 (23.7)	
Third	1026 (26.0)	5844 (25.8)	
Lowest	1088 (27.6)	6561 (29.0)	
Exhaustion			
Mean (SD)	11.84 (3.62)	11.48 (3.61)	<.001^b^
Quartile counts			
Highest	866 (21.9)	4321 (19.1)	<.001^c^
Second	1202 (30.5)	6660 (29.4)	
Third	864 (21.9)	5093 (22.5)	
Lowest	1015 (25.7)	6546 (28.9)	
Disengagement			
Mean (SD)	9.24 (3.56)	9.36 (3.58)	.047^b^
Quartile counts			
Highest	872 (22.1)	5574 (24.6)	.005^c^
Second	949 (24.0)	5381 (23.8)	
Third	962 (24.4)	5182 (23.0)	
Lowest	1164 (29.5)	6483 (28.7)	
**Learning environment**			
Emotional climate			
Mean (SD)	9.80 (3.29)	9.62 (3.21)	.002^b^
Quartile counts			
Highest	683 (17.3)	3406 (15.1)	<.001^c^
Second	1175 (29.8)	6553 (29.0)	
Third	1010 (25.6)	6299 (27.8)	
Lowest	1079 (27.3)	6362 (28.1)	
Student-faculty interaction			
Mean (SD)	14.09 (3.45)	14.29 (3.35)	<.001^b^
Quartile counts			
Highest	952 (24.1)	5655 (25.0)	.002^c^
Second	544 (13.8)	3132 (13.8)	
Third	1273 (32.2)	7721 (34.1)	
Lowest	1178 (29.8)	6112 (27.0)	

Abbreviation: URIM, underrepresented in medicine.

^a^ The unadjusted model includes only URIM status and the indicated burnout component. The adjusted model includes Medical Student Learning Environment Survey components broken into quartiles for emotional climate and student-faculty interaction. The fully-adjusted model includes the Medical Student Learning Environment Survey components, age, sex, debt burden, marital status, and school ownership.

^b^ *P* value represents *t* test.

^c^ *P* value represents Pearson χ^2^ test.

Our unadjusted model for overall burnout demonstrated no difference in the odds of being in the top quartile for overall burnout between students who are URIM and those who are not (OR, 1.03 [95% CI, 0.95-1.12]). However, students who are URIM had higher odds of being in the top quartile in the exhaustion model (OR, 1.19 [95% CI, 1.09-1.29]) and had lower odds of being in the top quartile for disengagement (OR, 0.87 [95% CI, 0.80-0.94]) compared with students who are not URIM (Table 3). These trends remained consistent when adjusted for MSLES and in the fully adjusted model. The interaction term for the association between URIM status and MSLES was not significant. Notably, students who reported experiencing a discrimination event related to their race and ethnicity had significantly higher odds of experiencing overall burnout (OR, 1.43 [95% CI, 1.30-1.58]).

**Table 3.** Logistic Regression Model Assessing the Association Between URIM Status and Burnout

Characteristic	OR (95% CI)								
	Unadjusted			Adjusted for MSLES			Fully adjusted		
	Top in overall burnout	Top in disengagement	Top in exhaustion	Top in overall burnout	Top in disengagement	Top in exhaustion	Top in overall burnout	Top in disengagement	Top in exhaustion
Not URIM	1 [Reference]	1 [Reference]	1 [Reference]	1 [Reference]	1 [Reference]	1 [Reference]	1 [Reference]	1 [Reference]	1 [Reference]
URIM	1.03 (0.95-1.12)	0.87 (0.80-0.94)	1.19 (1.09-1.29)	1.06 (0.97-1.15)	0.86 (0.79-0.94)	1.22 (1.12-1.33)	1.02 (0.93-1.12)	0.86 (0.79-0.95)	1.15 (1.05-1.26)
MSLES quartile: emotional climate									
Top	1 [Reference]	1 [Reference]	1 [Reference]	1 [Reference]	1 [Reference]	1 [Reference]	1 [Reference]	1 [Reference]	1 [Reference]
Second	NA	NA	NA	1.66 (1.42-1.94)	1.81 (1.56-2.12)	0.94 (0.84-1.08)	1.69 (1.44-1.98)	1.87 (1.61-2.17)	0.96 (0.84-1.09)
Third	NA	NA	NA	3.16 (2.71-3.70)	3.54 (3.06-4.11)	1.53 (1.35-1.75)	3.20 (2.74-3.75)	3.65 (3.15-4.24)	1.52 (1.34-1.74)
Bottom	NA	NA	NA	9.32 (7.99-10.90)	9.92 (8.55-11.55)	3.73 (3.27-4.26)	9.36 (8.01-10.98)	10.22 (8.81-11.91)	3.68 (3.22-4.21)
MSLES quartile: student-faculty interaction									
Top	1 [Reference]	1 [Reference]	1 [Reference]	1 [Reference]	1 [Reference]	1 [Reference]	1 [Reference]	1 [Reference]	1 [Reference]
Second	NA	NA	NA	1.07 (0.94-1.22)	1.16 (1.02-1.31)	1.07 (0.93-1.22)	1.05 (0.92-1.20)	1.14 (1.01-1.29)	1.04 (0.91-1.19)
Third	NA	NA	NA	1.23 (1.11-1.37)	1.27 (1.15-1.41)	1.38 (1.24-1.53)	1.21 (1.08-1.34)	1.25 (1.13-1.38)	1.35 (1.21-1.50)
Bottom	NA	NA	NA	1.79 (1.60-2.01)	1.92 (1.72-2.14)	1.82 (1.62-2.04)	1.71 (1.52-1.92)	1.84 (1.65-2.05)	1.73 (1.54-1.94)
Discrimination event	NA	NA	NA	NA	NA	NA	1.43 (1.30-1.58)	1.35 (1.23-1.49)	1.48 (1.34-1.63)

Abbreviations: MSLES, Medical Student Learning Environment Survey; OR, odds ratio; URIM, underrepresented in medicine.

A dose-dependent association was observed between the MSLES quartiles and burnout for both emotional climate and student-faculty interaction, with students in the lower quartiles having greater odds of burnout (Table 3). Medical students in the bottom quartiles of both MSLES categories, student-faculty interactions (OR, 1.71 [95% CI, 1.52-1.92]) and emotional climate (OR, 9.36 [95% CI, 8.01-10.98]), reported significantly higher rates odds of burnout. This association is particularly strong for the emotional climate with more than a 9-fold increase in the odds of burnout compared with those in the top quartile (OR, 9.36 [95% CI, 8.01-10.98]) (Table 3).

## Discussion

Our results demonstrate that overall burnout among students who are URIM vs those who are not is similar while students who are URIM were more likely to have high scores in exhaustion-related burnout and were less likely to have high disengagement-related burnout compared with students who are not URIM. Our finding related to overall burnout is consistent with previous literature that used the Maslach Burnout Inventory and demonstrated similar burnout scores between students who are URIM and those who are not.^6,33^ Garcia et al^18^ also used the Maslach Burnout Inventory and demonstrated that comparative burnout scores, in terms of emotional exhaustion and depersonalization, were similarly insignificant among a group of physicians. By using the OLBI-MS, our study adds to the literature regarding burnout among medical students from minoritized and marginalized racial and ethnic groups by elucidating emotional/physical exhaustion and disengagement in the pursuit of medicine alongside an assessment of the students’ learning environment with the MSLES, offering additional insights associated with burnout not included in the Maslach Burnout Inventory.^6,18,36,37^

Students who are URIM were more likely to report symptoms of cognitive and physical exhaustion in our study but less likely to report symptoms of disengagement. We found that students who are URIM experience discrimination at higher rates than peers who are not URIM, and having experienced discrimination was associated with students’ increased odds of reporting burnout. These findings suggest that burnout can and does vary among medical students based on their own unique experiences and adversity faced within the medical school environment. This discrepancy of burnout type has not yet been documented in prior literature and suggests that burnout may be experienced differentially among students who are URIM.

One possible explanation for this differential experience of burnout is that concurrent with a steady increase in diversity-related initiatives in medical schools throughout the past several years, engagement among students who are URIM in the medical school environment may indeed be increased.^38,39,40,41^ This may paradoxically lead to exhaustion-related burnout due to a *minority tax*, defined as a multifaceted entity that encompasses the extra burdens that physicians and trainees who are URIM often face during their medical career. The minority tax can involve feelings of isolation, a lack of identity-aligned mentorship, and unequal responsibility in diversity initiatives.^42,43,44^ While faculty who are URIM are driven to serve their own communities and are thus engaged, they may feel disproportionately required to spearhead efforts related to diversity compared with colleagues who are not URIM, causing exhaustion-related burnout.^42,44,45^ Additionally, it has been reported that many faculty who are URIM feel that these important initiatives go unrewarded and lack faculty support from those who are not URIM, which may further lead to exhaustion-related burnout.^42,44^ Herein, our study suggests that this pattern of burnout among individuals who are URIM, whereby medical students who are URIM are engaged with their medical school community and curriculum but experience high levels of exhaustion, may occur even earlier in the medical career pathway (prior to the faculty level).^6^ Medical students who are URIM are more likely to face additional responsibilities and hardships outside of their medical school activities that could contribute to their elevated experience of cognitive and physical exhaustion.^46^

Second, our findings demonstrate that differential perceptions in the medical school learning environment exist between medical students who are URIM and those who are not. Students who are URIM are more likely to report less positive student-faculty interactions, yet students who are URIM are more likely to report higher levels of emotional climate in the learning environment. This discrepancy could be explained by the framing of the survey questions, which highlight specifically interactions with faculty members vs a student’s general experience in medical school, which is influenced more greatly by ones’ peers. Our model showed that poorer perceptions of the learning environment was strongly associated with increased levels of burnout irrespective of URIM status. This was particularly strong for the emotional climate aspect of the learning environment. In terms of student-faculty interactions, our findings are concurrent with prior literature that suggests students who are URIM struggle to find identity-aligned mentors.^38^ Additionally, discrimination by faculty in the form of lower evaluations or being denied opportunities may play a significant role in contributing to lower perceptions of student-faculty interactions among students who are URIM, particularly because our study demonstrated that discrimination events based on race or ethnicity occur at a much greater level for students who are URIM.^19,47^

It may appear contradictory that medical students who are URIM experience more negative events and perceive poorer student-faculty interactions yet are less likely to become disengaged with the profession. One potential reason for this is a high level of resiliency and dedication to medicine among medical students who are URIM. Several research groups have posited that increased obstacles and different life experiences may promote resiliency among medical students who are URIM compared with peers who are not URIM.^6,36^ Another potential explanation is survival bias; it is possible students who are URIM faced greater obstacles on their way to medical school, ensuring that fewer students make it to and through medical school.^48^

Medical students who are URIM experience more episodes of discrimination and are at greater risk for exhaustion-related burnout, which may only increase as one transitions into resident and then faculty levels.^18,49,50^ As such, it is necessary for medical school administration and national governing bodies to ameliorate the higher risk of exhaustion-related burnout among medical students who are URIM. Addressing the minority tax is one potential avenue. Previous work among faculty who are URIM has demonstrated that, although faculty who are URIM personally believe in the mission of diversification, they often feel undervalued and undersupported for their efforts.^45,51^ It is reasonable that this sentiment might similarly be mirrored among medical students engaged in diversity initiatives. As such, medical schools must offer robust administrative, financial, and faculty support to students who are URIM who are engaged in diversity-related initiatives. Second, there must be broader support from medical students who are not URIM to play significant roles in diversity and inclusion initiatives. For example, some institutions offer paid fellowships, certificate programs, and stipends to support medical students pursuing diversity-related work.^52,53^

Additionally, medical students who are URIM are more likely to report poor perceptions of the medical school learning environment, further increasing one’s likelihood of reporting high levels of burnout. Expanding faculty who are URIM in key administrative positions may allow for these individuals to more easily serve as mentors and role models.^54,55^ Moreover, it is critical to not only increase the number of faculty who are URIM, but to provide these faculty with the necessary support to be effective and successful mentors.^38,56^ Last, racial discrimination, particularly toward medical students who are URIM, must be addressed. Medical schools must implement zero-tolerance policies for racism among students and faculty in the classroom and in clinical settings, implement longitudinal bias trainings, and increase mental health support for students who encounter discrimination and mistreatment based on race.

### Limitations

This analysis has several important limitations that warrant further exploration in future studies. As previously mentioned, the metrics for burnout focused on exhaustion and disengagement, while the learning environment metric similarly focused on emotional climate and student-faculty interactions. While these are validated measurements, it is possible they are unable to completely capture all factors that influence burnout. For example, the intersectionality of multiple marginalized identities, including sex and sexual orientation, could not be adequately accounted for in this analysis. Further research that expands the assessment of these different factors and their association at the point of both data collection and analysis will be crucial to properly address burnout in intersectional populations. Another potential limitation is the possibility of burnout predictors that could not be controlled for, including socioeconomic status, social isolation, and racial discrimination beyond the confines of the medical school training. Finally, given the nature of the survey, it is possible that there is an element of survival bias (ie medical students who are URIM who are graduating medical school demonstrate high levels of resiliency compared with the average population). Owing to the timing of the survey, recall bias may also inform reported experiences of burnout and longitudinal methods of assessing burnout are needed.

## Conclusions

Overall burnout among students who are URIM and those who are not was similar; however, medical students who are URIM report more episodes of discrimination, greater rates of exhaustion-related burnout, and lower positive perceptions of student-faculty interactions in the medical school learning environment compared with their counterparts who are not URIM. To facilitate the well-being and academic successes of medical students who are URIM, institutions must invest in evaluating their learning environments to ameliorate root causes of discrimination, mistreatment, and undue burdens placed on students who are URIM.

## Notes

Supplement. **eTable.** Burnout and MSLES Questions Included in the AAMC-GQ **eFigure.** Inclusion and Exclusion of Responses to the 2016 and 2017 AAMC Graduation Questionnaire
