# Supplementary material for: Perceptions on Burnout and the Medical School Learning Environment of Medical Students Who Are Underrepresented in Medicine
Source: JAMA Netw Open. 2022 Feb 23;5(2):e220115. doi: 10.1001/jamanetworkopen.2022.0115 (PMC8867243; doi:10.1001/jamanetworkopen.2022.0115)
Supplement: Supplement. — eTable. Burnout and MSLES Questions Included in the AAMC-GQ eFigure. Inclusion and Exclusion of Responses to the 2016 and 2017 AAMC Graduation Questionnaire [file jamanetwopen-e220115-s001.pdf]

Supplemental Online Content

O'Marr JM, Chan SM, Crawford L, Wong AH, Samuels E, Boatright D. Perceptions on burnout and the medical school learning environment of medical students who are underrepresented in medicine. *JAMA Netw Open*. 2022;5(2):e220115. doi:10.1001/jamanetworkopen.2022.0115

eTable. Burnout and MSLES Questions Included in the AAMC-GQ
eFigure. Inclusion and Exclusion of Responses to the 2016 and 2017 AAMC Graduation Questionnaire

This supplemental material has been provided by the authors to give readers additional information about their work.

© 2022 O'Marr J et al. *JAMA Network Open.*

eTable. Burnout and MSLES Questions Included in the AAMC-GQ

**Burnout**: "Please indicate the extent to which you agree with the following statement" (0: Strongly Disagree, 1: Disagree, 2: Agree, 3: Strongly Agree)	
**Disengagement**	**Exhaustion**
1. I always find new and interesting aspects in my medical school work [a]	1. There are days when I feel tired before I arrive at medical school
2. It happens more and more often that I talk about my medical school work in a negative way	2. After a day of medical school, I tend to need more time than in the past in order to relax and feel better
3. I can tolerate the pressure of my medical school work very well [a]	3. Lately, I tend to think less at medical school and do my medical school work almost mechanically
4. I find my medical school work to be a positive challenge [a]	4. During my medical school work, I often feel emotionally drained
5. Over time, one can become disconnected from medical school work	5. After a day of medical school, I have enough energy for my leisure activities [a]
6. Sometimes I feel sickened by my medical school work.	6. After a day of medical school, I usually feel worn out and weary
7. The study of medicine is the only thing that I can imagine myself doing [a]	7. Usually, I can manage the amount of my medical school work well [a]
8. I feel more and more engaged in my medical school work [a]	8. When I am at medical school, I usually feel energized [a]
**MSLES:** Think about HOW OFTEN you experience the following at your medical school. Determine your response by choosing one of the categories of frequency given below. Choose the category that best approximates your perceptions"(0: Never, 1: Almost Never, 2: Sometimes, 3: Fairly Often, 4: Very often, 5: Always)	
**Emotional Climate**	**Student-Faculty Interactions**
1. The educational experience makes students value themselves	1. When giving criticism or answering a question, faculty are genuinely interested in helping the student
2. The educational experience makes students feel a sense of achievement	2. Faculty and administrators give personal help to students having academic difficulty
3. The educational experience makes students feel confident of their academic abilities	3. Faculty are reserved and distant with students [a]
	4. The educational experience makes students value themselves

[a] Indicates item was reverse coded for the analysis.

© 2022 O'Marr J et al. *JAMA Network Open.*

eFigure. Inclusion and Exclusion of Responses to the 2016 and 2017 AAMC Graduation Questionnaire. GQ= Graduation Questionnaire AAMC = Association of American Medical Colleges. MSLES = Medical Student Learning Environment Survey. OLBQI = Oldenburg Burnout Inventory for Medical Students

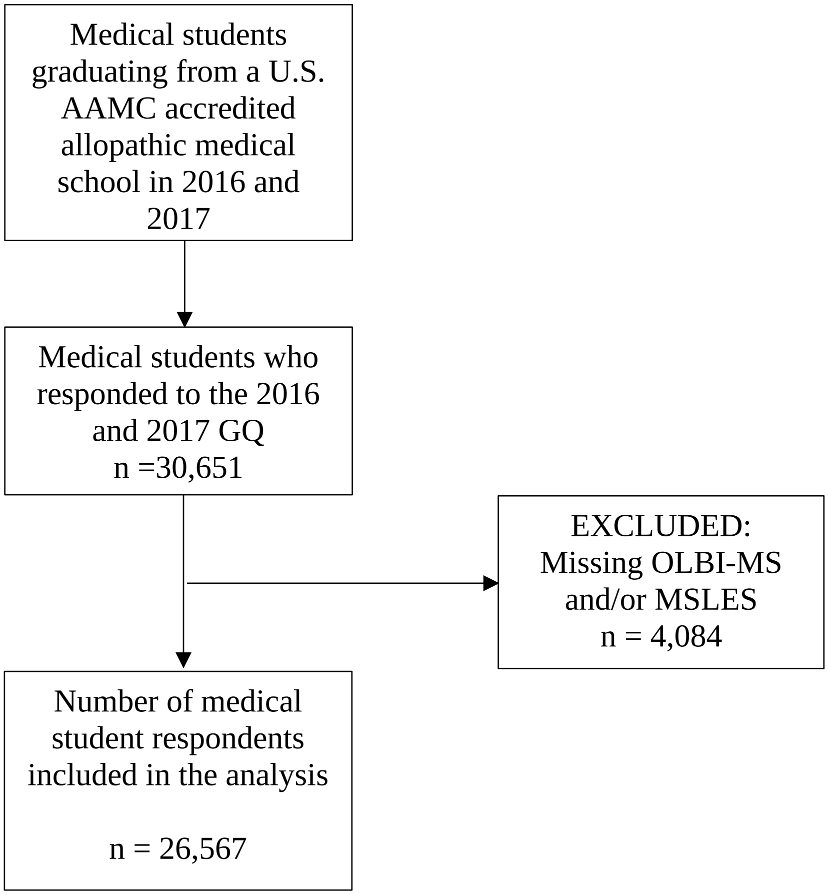

© 2022 O'Marr J et al. *JAMA Network Open.*
